# Comparative effectiveness and safety of antiviral agents for patients with COVID-19: Protocol for a systematic review and individual-patient data network meta-analysis

**DOI:** 10.1371/journal.pone.0241947

**Published:** 2020-11-09

**Authors:** Yulong Cao, Jiao Shan, Zhizhong Gong, Jiqiu Kuang, Yan Gao

**Affiliations:** 1 Department of Hospital-Acquired Infection Control, Peking University People's Hospital, Beijing, The People's Republic of China; 2 Department of Hospital-Acquired Infection Control, Beijing Jishuitan Hospital, Beijing, The People's Republic of China; 3 Division of Medical Affairs, Beijing Hospital of Traditional Chinese Medicine, Capital Medical University, Beijing, The People's Republic of China; 4 Institute for Hospital Management of Tsinghua University, Beijing, The People's Republic of China; BronxCare Health System, Affiliated with Icahn School of Medicine at Mount Sinai, UNITED STATES

## Abstract

**Background:**

A recent cluster of pneumonia cases in Wuhan, China, has been caused by severe acute respiratory syndrome coronavirus 2 (SARS-CoV-2). We propose the protocol described below to perform an individual-patient data (IPD) network meta-analysis (NMA) in order to evaluate the efficacies of different antiviral drugs to treat patients with coronavirus disease 2019 (COVID-19).

**Materials and methods:**

We will search the Medline, EMBASE, Cochrane Library, SinoMed, CNKI and VIP databases from their inceptions through July 2020. There will be no restrictions on language, publication year, or publication type. Randomized clinical trials (RCTs) and prospective cohort studies with antiviral treatments for COVID-19 will be considered. Two reviewers will independently select studies and collect data. Risk-of-bias assessments will be completed using the Cochrane risk-of-bias scale. Primary outcome will be the COVID-19 recovery rate. We will combine aggregated data from IPD with the NMA in a single model, compare the effects of different antiviral drugs on patient-relevant efficacy, and rank the results to decide which is the most effective.

**Trial registration:**

PROSPERO registration number: CRD42020167038.

## Introduction

In December 2019, a cluster of cases of severe acute respiratory syndrome coronavirus 2 (SARS-CoV-2) -infected pneumonia emerged in Wuhan, Hubei, China. Although cases were originally identified as associated with exposure to the Huanan seafood market, the current epidemiological data indicate that there is person-to-person transmission of SARS-CoV-2 [1]. As of 1 May, 2020, a total of 3,344,435 cases have been reported, including 238,788 deaths [2]. At this writing, coronavirus disease 2019 (COVID-19) has propagated in hundreds of countries around the world. As the epidemic progresses, it causes serious damage to human health and places a substantial burden on the family, healthcare system, and society.

Coronaviruses are enveloped, non-segmented, positive-sense, single-stranded ribonucleic-acid (RNA) viruses, whose structures have characteristic club-shaped spikes. They are divided into four genera: α, β, γ, and δ. Although severe acute respiratory syndrome coronavirus (SARS-CoV), middle East respiratory syndrome coronavirus (MERS-CoV), and SARS-CoV-2 are all betacoronaviruses [3], they differ markedly in their genetic characteristics. SARS-CoV-2 has been proven to be a new type of coronavirus found in humans; there are currently no effective antiviral drugs.

According to the *Diagnosis and Treatment Protocol for Novel Coronavirus Pneumonia*, *(Trial Version 7)*, issued by the National Health Commission of the People’s Republic of China [4], interferon-alpha (IFN-α), lopinavir/ritonavir (LPV/r), ribavirin, chloroquine phosphate, arbidol and Xuebijing injection (a traditional Chinese patent medicine) are recommended as treatment based only on the anecdotal experience of experts; they are not supported by evidence-based medicine.

From treatment of symptoms at the beginning of the epidemic to clinical trials of antiviral drugs and updates, the treatment of COVID-19 is also advancing with the times and constantly improving. On January 23, 2020, patients with COVID-19 had reduced fever and fatigue after 12 hours of taking LPV/r. After 48 hours, the real-time reverse-transcriptase polymerase chain reaction (RT-PCR) results from oropharyngeal swabs (OPS) turned negative. However, in a trial of LPV/r involving patients with severe Covid-19, Cao et al. conclude that the administration of LPV/r did not result in a shorter time until clinical improvement, lower mortality, or lower SARS-CoV-2 RNA levels than standard care [5]. Some experts believe that the findings of Cao et al. cannot rule out the effectiveness of LPV/r, and have raised some doubts [6]. The first COVID-19 patient confirmed in the United States showed clinically significant improvements after intravenous infusion of remdesivir [7]. Many researchers are today exploring whether remdesivir is effective to COVID-19. On April 29, 2020, results of three important clinical trials of remdesivir in the treatment of COVID-19 were published on the same day. Hospitalized patients with advanced COVID-19 who received remdesivir had a 31% faster time to recovery than those who received placebo (p<0.001), according to a randomized, controlled trial (RCT) involving 1063 patients, which sponsored by the US National Institutes of Health [8]. Gilead Sciences, Inc. announced topline results from the open-label, Phase 3 SIMPLE trial. The study demonstrated that remdesivir may shorten the treatment course, increasing bed turnover rates [9]. Another remdesivir trial from China reported that remdesivir was not associated with statistically significant clinical benefits, adverse events in 102 (66%) of 155 remdesivir recipients versus 50 (64%) of 78 placebo recipients [10].

A network meta-analysis (NMA) model can estimate treatment effects even for treatments that have never been directly compared in a head-to-head study [11]. The optimal approach to assessing the transitivity assumption is using individual-patient data (IPD) across treatment comparisons, and IPD-NMA can tailor results to patient characteristics. It is currently unclear which treatment is optimal for COVID-19 patients. Therefore, we will perform an IPD-NMA to identify potential treatment effect modifiers and to estimate the safest and most effective treatments based on the clinical, laboratory, and radiological characteristics of COVID-19 patients.

## Materials and methods

This systematic review and IPD-NMA protocol has been registered on PROSPERO (CRD42020167038), and our manuscript will conform to the Preferred Reporting Items for Systematic reviews and Meta-Analysis Protocols (PRISMA-P) guidelines [12]. See S1 Table for the checklist.

### Ethics and dissemination

To the best of our knowledge, this study will be the first IPD-NMA examining the comparative safety and effectiveness of antiviral treatments for COVID-19. The IPD-NMA might be more powerful than the NMA using aggregated data; it will allow healthcare providers to individualize the management of patients with COVID-19. The findings of our study will fill an important knowledge gap in healthcare and can be used to inform clinicians, patients, and other decision makers.

The IPD-NMA does not require ethical approval, as it synthesizes data from clinical trials. We will request only anonymized data from authors, and we will link each patient to a specific identifier to prevent the patient from being identified.

The results of this systematic review and IPD-NMA will be of interest to stakeholders, including decision makers, guideline developers, clinicians, methodologists, and patients.

The dissemination of our findings will be knowledge user-driven and tailored to how and when knowledge users want to receive information. Team members will act as knowledge brokers using their networks—such as The Cochrane Collaboration, PRISMA-IPD, and the Drug Safety and Effectiveness Network (DSEN)—to facilitate dissemination.

### Search strategy

We will search the literature using the following databases: Medline, EMBASE, Cochrane Library, Chinese BioMedical Literature Database (SinoMed), Chinese National Knowledge Infrastructure (CNKI), and Chinese Science and Technology Periodical Database (VIP). There will be no beginning date identified, while the cut-off date will be 31 July 2020. The following search terms will be used individually or combined: “COVID-19,” “2019-nCoV,” “SARS-CoV-2,” “novel coronavirus pneumonia,” “IFN-α,” “lopinavir/ritonavir,” “chloroquine,” “hydroxychloroquine,” “ribavirin,” “arbidol,” “remdesivir,” “Xuebijing injection,” “randomized controlled trial,” and “cohort study.” We will also search the literature through the WHO International Clinical Trial Registry Platform (ICTRP) dataset for ongoing registered clinical trials and unpublished articles. Details of the search strategy in Medline are provided in S1 Appendix.

### Eligibility criteria

We will use Participant, Intervention, Comparator, Outcome, and Study design (PICOS) criteria. Eligible studies will be RCTs, non-randomized controlled trials and prospective cohort studies, including adults given different treatments that are compared with one another, best supportive care, or placebo. The specific PICOS criteria are as follows:

#### Participants

Adults (aged ≥18 years) at the time of signing the informed-consent form. RT-PCR positive for SARS-CoV-2 from analysis of nasopharyngeal or oropharyngeal swab specimens obtained; lung involvement confirmed with chest imaging.

#### Interventions/Comparators

IFN-α, LPV/r, remdesivir, chloroquine, ribavirin, arbidol, Xuebijing injection, a range of potentially effective antiviral drugs alone or in any combination, and placebo.

#### Outcomes

Primary-outcome: Time to clinical recovery (TTCR; timeframe: up to 28 days). TTCR is defined as the time (in days) from initiation of study treatment (active or placebo) until normalization of fever, respiratory rate, oxygen saturation, and alleviation of cough, sustained for at least 72 hours. Normalization and alleviation criteria: fever, ≤36.6°C axillary, ≤37.2°C oral, or ≤37.8°C rectal or tympanic; respiratory rate, ≤24/minute on room air; oxygen saturation, >94% on room air; cough, mild or absent on a patient-reported scale of severe, moderate, mild, or absent. In addition, the patient must agree not to enroll in another study prior to completion of this one. Time to RT-PCR test results for SARS-CoV-2 RNA turned negative.

Secondary-outcome: All-cause mortality, baseline pulse oxygen saturation (SpO2) during screening, partial pressure of oxygen (PaO2)/ Fraction of inspiration O_2_ (FiO2) <300 mmHg or a respiratory rate ≥24 breaths per min without supplemental oxygen; frequency of respiratory progression, defined as SpO2 ≤94% at room temperature” here or PaO2/FiO2 <300 mmHg and requirement for supplemental oxygen or more-advanced ventilator support; time to defervescence (in those with fever at enrollment); time to cough reported as mild or absent (in those with cough at enrollment rated severe or moderate); time to dyspnea reported as mild or absent (on a scale of severe, moderate, mild, or absent in those with dyspnea at enrollment rated as severe or moderate); frequency of requirement for supplemental oxygen or non-invasive ventilation; time to SARS-CoV-2 RT-PCR negative in upper-respiratory-tract (URT) specimen; change (reduction) in SARS-CoV-2 viral load in URT specimen as assessed by area under the viral-load curve; frequency of requirement for mechanical ventilation (divided in invasive and non invasive ventilation); and frequency of serious adverse events.

#### Study designs

We will include RCTs and quasi-experimental studies (pre- and non-RCTs) irrespective of the methods used to allocate patients to groups. Consideration of ethical or safety issues associated with the application of unknown treatment and prospective cohort studies will also be included.

### Study selection

We will use Endnote X9 software (Clarivate Analytics, Philadelphia, PA USA) to exclude duplicate studies from different databases, and then to exclude obviously unrelated documents based on titles and abstracts. The full text of every potentially relevant study that is retrieved will be obtained, and two of the reviewers (CYL and SJ) will scrutinize these reports independently to determine which require further assessment. Differences in eligibility assessments will be resolved by discussion, and, when necessary, a final consensus will be reached with the assistance of a third reviewer.

### Data extraction

Training in data extraction and reviewer examination will be conducted in advance to guarantee quality of extraction. We will use a standardized checklist to extract data for our IPD-NMA from studies that meet the inclusion criteria. The following variables will be extracted for each study: study characteristics (first author, year of publication, country of publication, institution, and sponsor), study characteristics (sample size, study design, number of groups, methodology of randomization and allocation concealment, blinding, and settings), patient characteristics (e.g., gender, age, and diagnosis), interventions and controls (e.g., type of intervention, frequency and duration of treatment or follow-up), outcomes (e.g., type of outcome, definition of outcome, and timepoint of assessment), and results (statistical description of outcomes such as mean, standard deviation (SD), observed and total sample sizes, and adverse events). When relevant details are missing from articles, we will attempt to contact (by email) the relevant authors to obtain the missing information. Rechecking by and discussion with a third reviewer will be required if any disagreement occurs during data extraction. We will inform all authors that their articles will be appropriately cited, and, if they agree, they will be acknowledged in our paper.

### Acquisition of IPD data

A potential difficulty in the conduct of our study is that IPD can only be obtained by contacting the original trial authors. To overcome this difficulty and improve the response rate, we will only request anonymised data from the authors, and use validated approaches suggested for electronic surveys and provide a cash incentive to each author.

### Risk-of-bias and quality appraisal

Two reviewers will independently evaluate the risk of bias in each included study after pilot testing a random sample of three studies. Disagreements will be resolved by discussion. We will (1) compare IPD provided by the investigator with aggregate data reported in the publication, (2) assess whether the eligibility criteria of each study are in agreement with the IPD [13], and (3) draw a comparison-adjusted funnel plot that allows for examination of heterogeneity, selective reporting, and publication and funding biases. If significant asymmetry is found after a visual inspection of the funnel plot, meaning that publication bias will be suspected, we will include a statement in our results section and our summary-of-findings table, with a corresponding note of caution in our discussion section.

We have adopted the Cochrane risk-of-bias tool to evaluate risk of bias for each included RCT with regard to the following items: randomization allocation; randomization concealment; blinding of participants and personnel; blinding of outcome assessment; data integrity; selective-reporting bias; and other biases such as trial design, baseline similarity of groups, and early cessation of treatment. Each potential item will be graded as having a high, low, or unclear level of bias; and we will provide a quote from the study report together with a justification for our judgment in the risk-of-bias table. We will also independently assess the quality of evidence in each NMA using the Grades of Recommendation, Assessment, Development, and Evaluation (GRADE) approach as adapted for NMA.

### Data analysis

We will perform a Bayesian NMA for each treatment comparison, and then present summary statistics and potential outlier patient values to describe the outcome data in each study. All of the IPD from included studies will be combined into a multilevel model in which each study will constitute a different cluster. We will use odds ratios for categorical variables and mean difference effect sizes for continuous variables.

We will perform a two-part stage analysis with the same between-study variance in both parts and accounting for treatment × covariate interactions. The first part will entail a 1-stage model using IPD only, while the second will entail applying a NMA modeling aggregate data to assess the safety and effectiveness of different antiviral drugs for NCP. We will deal with missing data using multiple imputations.

NMA will be performed using the rjags package in the R statistical software package, version 3.6.2. Just Another Gibbs Sampler (JAGS) is a software program for the statistical analysis of Bayesian hierarchical models using the Markov chain Monte Carlo (MCMC) method. Parameters for JAGS will be as follows: number of chains = 4, tuning iterations = 20,000, simulation iterations = 50,000, thinning interval = 10, inference samples = 10,000, and variance scaling factor = 2.5. We will use the Brooks–Gelman–Rubin method to assess convergence. In this process, a potential scale reduction factor (PSRF) is calculated by comparing within-chain and between-chain variance. Bayesian methods will also be used with both fixed-effect and random-effect multiple-treatment comparisons (MTCs) for indirect comparisons. Cumulative probability will be plotted to help identify the best method in each treatment arm for each outcome (rankograms) in graphical form (Surface Under the Cumulative Ranking curve [SUCRA]). An inconsistency model and a consistency model will be fitted to the data, and model selection will be based on deviance information criteria. Either a 95% confidence interval (CI) including 1 in the loop analysis or a *P*-value of <0.05 in the node-splitting analysis will be considered to indicate a significant inconsistency.

### Sensitivity analysis

To explore possible reasons for heterogeneity and to assess the robustness of the pooled results, we will undertake sensitivity analysis to comparing studies with a low risk of bias to those with high or unclear risk of bias. In addition, if low-quality literatures bring significant heterogeneity, they are going to be excluded, evaluate the sensitivity again to ensure the homogeneity of study.

## Supporting information

S1 Table PRISMA-P checklist. (DOC)

S1 Appendix Search strategy for medline. (DOCX)
