# Supplementary material for: Comparative effectiveness and safety of antiviral agents for patients with COVID-19: Protocol for a systematic review and individual-patient data network meta-analysis
Source: PLoS One. 2020 Nov 9;15(11):e0241947. doi: 10.1371/journal.pone.0241947 (PMC7652299; doi:10.1371/journal.pone.0241947)
Supplement: S1 Appendix — (DOCX) [file pone.0241947.s002.docx]

**S1 Appendix. Search Strategy for Medline.**

("COVID-19"[Mesh] OR "2019-nCoV"[Mesh:NoExp] OR "severe acute respiratory syndrome coronavirus 2"[Mesh] OR "SARS-CoV-2"[Mesh] OR "Wuhan coronavirus"[Mesh] OR 2019 novel coronavirus*[tw] OR coronavirus disease 2019 virus*[tw] OR COVID19 virus*[tw] OR 2019-nCoV infection[tw] OR novel coronavirus pneumonia*[tw])

AND

chlorochin*[tw] OR aralen*[tw] OR hydroxychloroquine*[tw] OR oxychloroquine*[tw] OR chloroquine phosphate*[tw] OR chingamin phosphate*[tw] OR interferon-alpha*[tw] OR alpha interferon*[tw] OR IFN-α*[tw] OR lopinavir-ritonavir drug combination*[tw] OR lopinavir/ritonavir*[tw] OR LPV/r*[tw] OR ribavirin*[tw] OR rebetol*[tw] OR virazole*[tw] OR arbidol*[tw] OR umifenovir*[tw] OR remdesivir*[tw] OR xuebijing injection*[tw])

AND

("time to clinical recovery"[Mesh] OR "TTCR"[Mesh] OR "recovery time*"[Mesh] OR "all-cause mortality"[Mesh] OR "mortality*"[tw] OR "mortality rate*"[tw])

AND

("randomized controlled trial" [Publication Type] OR "randomized controlled trial" [All Fields] OR "RCT" [All Fields] OR "non-randomized controlled trial" [All Fields] OR "quasi-experimental studies" [All Fields] OR "cohort studies" [Publication Type] OR "prospective cohort studies" [All Fields])
